# Endoscopic thoracic facet joint denervation: A technical description and two cases

**DOI:** 10.1016/j.inpm.2026.100833

**Published:** 2026-11-03

**Authors:** Hasan Sen, Amanda N. Cooper, Aaron M. Conger, Joel M. Talsma, Mark A. Mahan, Zachary L. McCormick

**Affiliations:** a Department of Physical Medicine and Rehabilitation, University of Utah, Salt Lake City, UT, USA; b Biomedical Sciences, University of New England, Biddeford, ME, USA; c Department of Neurosurgery, Clinical Neurosciences Center, University of Utah, Salt Lake City, UT, USA

## Abstract

**Background:**

Recent dissection literature describes a common articular branch originating directly from the primary dorsal ramus or the proximal portion of the medial branch of the primary dorsal ramus of the spinal nerve, suggesting the possibility of new strategies for thoracic facet joint (TFJ) denervation. An endoscopic approach provides the ability to directly visualize and rapidly denude the entire inferior-lateral to superior-lateral aspect of the TFJ capsule while avoiding the exiting spinal nerve, ensuring complete transection of any articular branch from the primary dorsal ramus external to the neural foramen as well as any inconsistent articular branches from the medial branches prior to their termination in the TFJ capsule.

**Objective:**

Describe a novel technique for endoscopic TFJ denervation (ETFJD) and two case reports.

**Methods:**

Two patients who underwent ETFJD with our inferior-lateral to superior-lateral TFJ capsule-targeting technique were contacted. Numerical Rating Scale (NRS) pain intensity, Patient Global Impression of Change (PGIC), and patient satisfaction scores were collected by standardized telephone survey.

**Results:**

At 13.7 months, Patient 1 reported asymmetric improvement (left-sided NRS reduction from 9 to 2; right-sided NRS improvement from 9 to 7); PGIC 7, and satisfaction 5. Patient 2 reported NRS reduction from 9 to 0 at 17.9 months; PGIC 7, and satisfaction 5. No procedural complications occurred.

**Conclusion:**

We describe a novel technique for ETFJD and two case reports. Formal evaluation and optimization of this treatment strategy for refractory thoracic facet joint pain is warranted.

## 1 Introduction

Recently, strategies for thoracic facet joint (TFJ) denervation have evolved as newer dissection studies have revealed the presence of an articular branch arising from either the proximal portion of the medial branch or directly from the primary dorsal ramus of the spinal nerve, with greater variability observed at middle (T4–9) and lower (T10–12) thoracic levels [1], [2], [3], [4], [5], [6]. This proximal articular branch was not identified during legacy dissection work [7]; however, its consistent presence in more recent neuroanatomical studies suggests that a new paradigm for TFJ denervation may be possible. Given the prevalence of primary thoracic spine pain, the 2024 International Pain and Spine Intervention Society (IPSIS) Technical Manual and Atlas of Interventional Pain and Spine Procedures specifically called for this development [8].

The proximity of this articular branch to the spinal nerve before its termination in the TFJ capsule presents a challenge for safe and effective TFJ denervation using conventional radiofrequency ablation (RFA) techniques [3], [6]. While percutaneous multi-tined and bipolar RFA techniques have been proposed [6], [9], an endoscopic approach confers the ability to directly visualize and rapidly denude the entire inferior-lateral to superior-lateral aspect of the TFJ capsule while carefully avoiding the exiting spinal nerve. This ensures complete transection of the articular branch from the primary dorsal ramus external to the neural foramen, as well as any variant articular branches from the medial branches prior to their termination in the TFJ capsule.

Clinical outcome literature suggests that endoscopic neurectomy of lumbar medial branches can provide durable improvements in pain and disability in well-selected patients, and comparative data indicate long-term benefit over percutaneous medial branch RFA in some cohorts [10], [11]. However, technical descriptions and clinical outcomes specific to endoscopic TFJ denervation (ETFJD) remain limited. One retrospective case series of 15 patients has been reported, but this work included a vague description of the surgical technique for removal of facet joint capsular tissue, rendering reproducibility challenging [12].

Accordingly, the objectives of the present report were to (1) describe a technical approach for ETFJD and (2) present clinical outcomes in two patients treated with this procedure.

## 2 Technical description

### 2.1 Anatomical considerations

Recent cadaveric anatomical investigation of thoracic articular branches has demonstrated that zygapophyseal joint innervation may arise either from the dorsal ramus proper or from the proximal portion of the medial branch, with variation observed across thoracic levels (Fig. 1) [5]. In the mid thoracic spine (T4–8), articular branches have been shown to arise directly from the dorsal ramus prior to medial branch bifurcation rather than consistently from a distal medial branch [2]. Recent cadaveric investigation of mid thoracic levels (T6–9) has further demonstrated that thoracic articular branch nerves course along the superior aspect of the rib head just lateral to the zygapophyseal joint, where the superior aspect of the rib head may serve as an osseous landmark for fluoroscopic targeting [6]. At lower thoracic levels (T10–12), segment specific and transitional morphology has been shown to influence the course and topographic relationship of the medial branch relative to surrounding osseous landmarks [4]. Accordingly, the present technique was designed to permit direct visualization and transection of the neural structure identified along the expected course of any articular branch (whether originating directly from the dorsal ramus or from the medial branch) rather than relying solely on fixed fluoroscopic bony targets (Fig. 2).

**Fig. 1:**
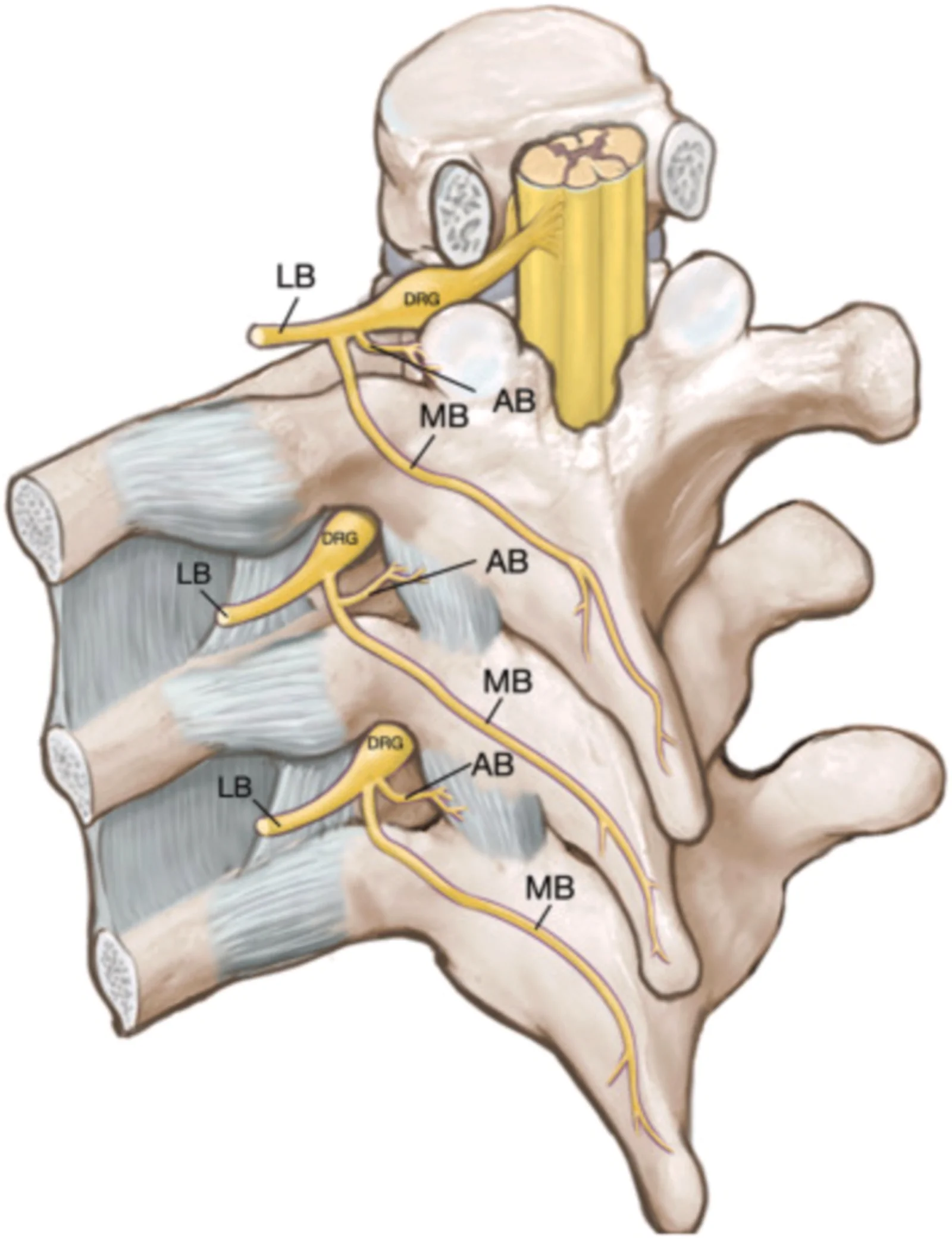
Schematic illustration of thoracic zygapophyseal joint innervation demonstrating two known variations in the origin of the articular branch, which arises directly from the dorsal ramus (first and third spinal nerve levels depicted) or from the proximal portion of the medial branch (second spinal nerve level depicted). Figure reproduced with permission from Talsma JM, Maus TP, Chitneni A, Hao D. Investigation of the anatomical course of articular branches to the thoracic zygapophysial joints. Interv Pain Med. 2024 Mar 11; 3(1):100399. doi: 10.1016/j.inpm.2024.100399. Abbreviations: AB = articular branch; DRG = dorsal root ganglion; LB = lateral branch; MB = medial branch.

**Fig. 2:**
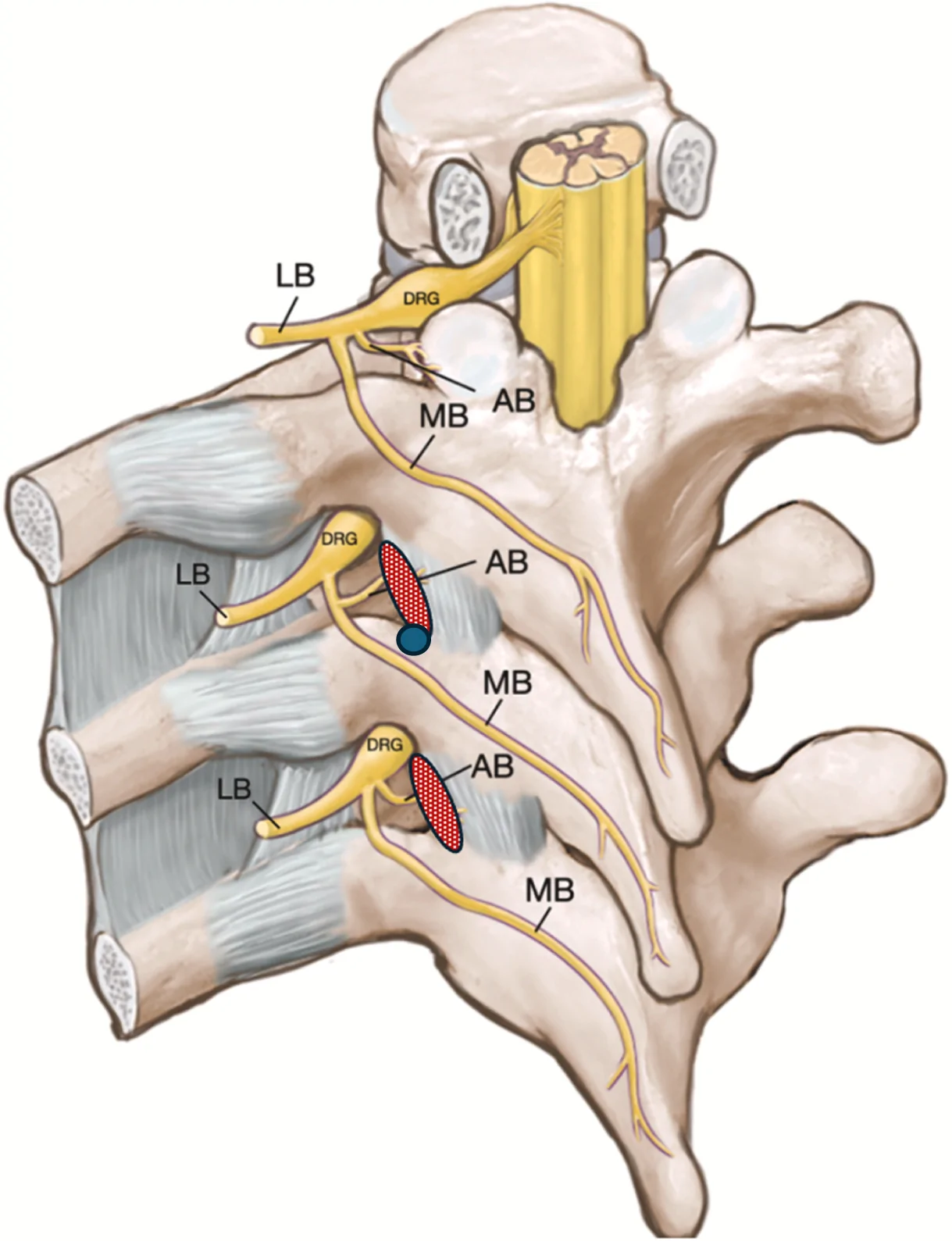
Schematic illustration of thoracic zygapophyseal joint innervation showing the initial docking position (dark blue circle) of the endoscope and the area (represented by ovals with red cross-hatching) wherein the entire lateral margin of the facet joint capsule is denuded using the proposed endoscopic approach, with transection of the articular branch. Note how the area of denudation includes distal fibers of the articular branch regardless of its origin. Figure reproduced with permission from Talsma JM, Maus TP, Chitneni A, Hao D. Investigation of the anatomical course of articular branches to the thoracic zygapophysial joints. Interv Pain Med. 2024 Mar 11; 3(1):100399. doi: 10.1016/j.inpm.2024.100399. Abbreviations: AB = articular branch; DRG = dorsal root ganglion; LB = lateral branch; MB = medial branch. (For interpretation of the references to colour in this figure legend, the reader is referred to the Web version of this article.)

### 2.2 Thoracic medial branch blocks

Diagnostic medial branch blocks (MBBs) were performed on two separate occasions using injections of 0.5 mL of 4% lidocaine and 0.5 mL of 0.5% bupivacaine, respectively. A positive block response was defined as ≥80% pain relief that was consistent with the expected duration of the anesthetic used.

### 2.3 Procedural technique

ETFJD procedures were performed with the patient under monitored anesthesia care. Using fluoroscopic anteroposterior (AP) imaging, the endoscope docking position was identified at the inferior-lateral aspect of the targeted facet joint, where the superior articular process and transverse process of the caudal vertebra meet the inferior articular process of the cephalad vertebra (blue circle, Fig. 2). An AP view aligned with the superior endplate of the vertebral body closest to the docking position was obtained to provide appropriate visualization for safe initial docking, and the corresponding skin entry site was marked. After subcutaneous infiltration with a small amount of 1% lidocaine with epinephrine, a 22 G 3.5-inch spinal needle was advanced to the docking position (Fig. 3A), where approximately 2 mL of 1% lidocaine with epinephrine was injected to anesthetize the deep peri-facet joint structures; an additional 2 mL was injected during needle withdrawal to anesthetize the entire planned endoscopic access pathway.

**Fig. 3:**
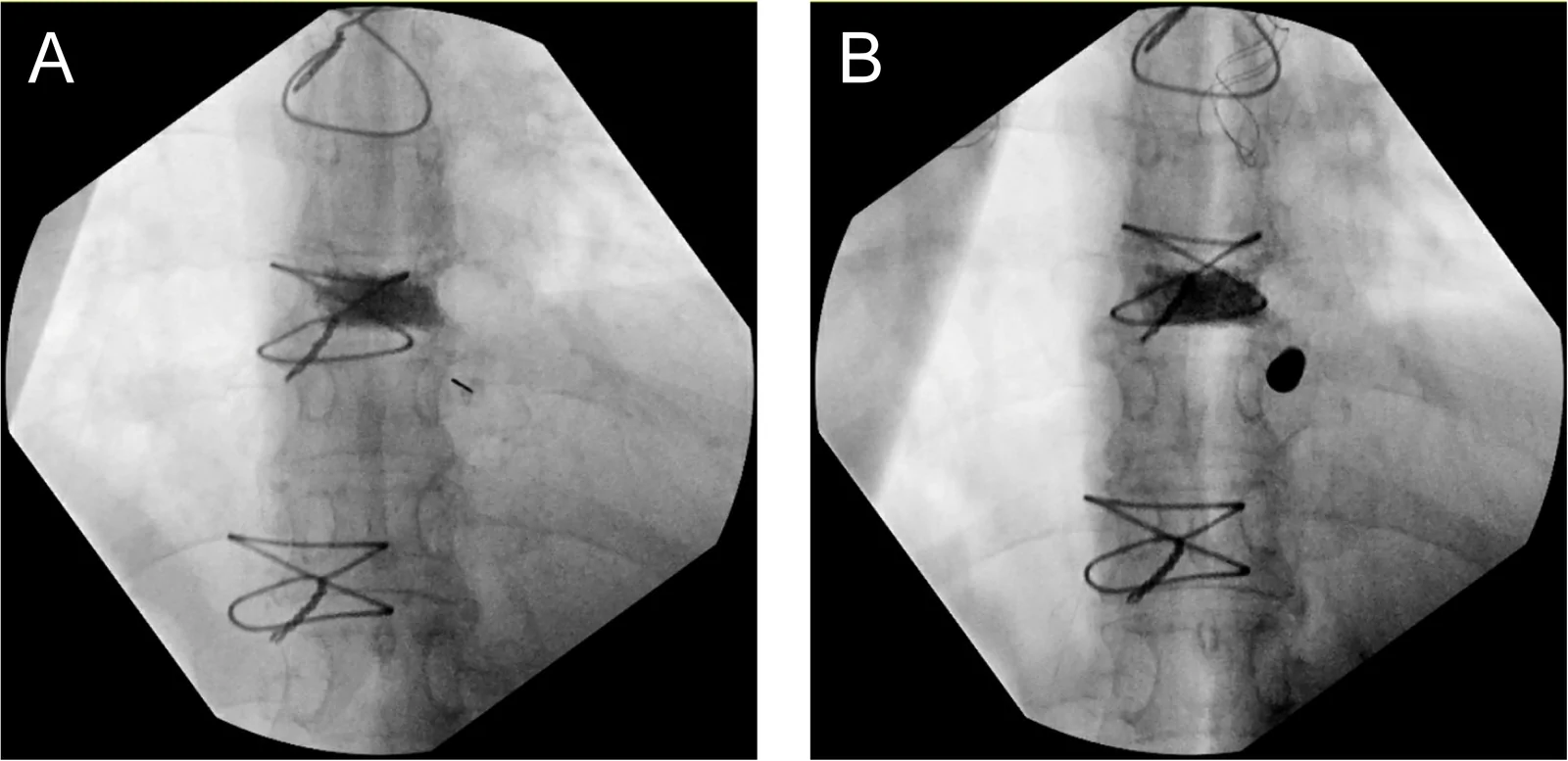
Anteroposterior (AP) fluoroscopic views of the thoracic spine demonstrating: **(A)** Spinal needle placement at the junction of the T9 transverse process and the inferior-lateral margin of the right T8–9 facet joint. **(B)** Tissue dilator placement in initial docking position at the junction of the T9 transverse process and the inferior-lateral margin of the right T8–9 facet joint.

An 8-mm skin incision was created with an #11 blade. A tissue dilator was placed through the incision and advanced under fluoroscopic guidance to the docking position (Fig. 2, Fig. 3B). A tubular retractor was guided along the tissue dilator to its distal aspect, and then the tissue dilator was removed. A 7-mm uniportal endoscope was then introduced through the working tube for illumination and magnified visualization. Bipolar cautery was used to dissect soft tissues and coagulate small blood vessels as needed until any neural structure consistent with the thoracic medial branch or a direct proximal articular branch was directly visualized. The nerve was then transected using either a pituitary or scissor instrument. Next, the lateral aspect of the facet joint was denuded from its inferior-lateral to superior-lateral margin (Fig. 4) using a combination of bipolar cautery and pituitary instruments. Notably, the thoracic medial branch or proximal articular branch may not always be reliably visualized; in such cases, denuding the lateral facet joint of its capsular tissue necessarily disrupts distal articular fibers, such that sensory denervation is possible without direct visualization of the neural tissue. Once only osseous aspects of the lateral aspect of the facet joint remained, the working area was suctioned and a small volume of corticosteroid (5–10 mg of triamcinolone acetate) diluted in 0.25% bupivacaine with epinephrine was instilled through the endoscopic irrigation port. The endoscope and the working tube were removed, and this process was repeated at adjacent spinal levels of interest depending on the planned number of facet joints included in the ETFJD procedure. Each skin incision was closed with an absorbable suture and covered with sterile dressings.

**Fig. 4:**
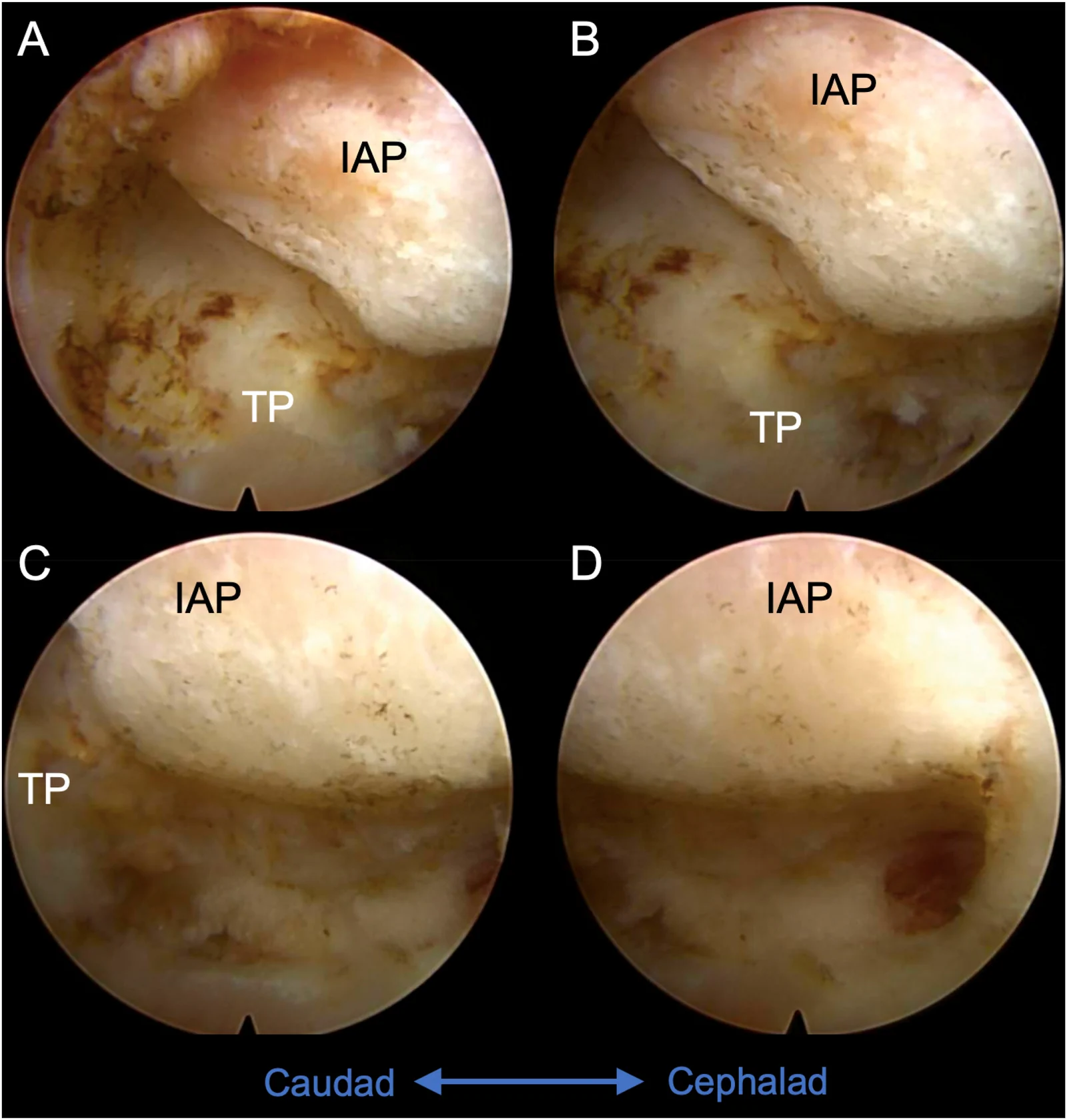
Endoscopic views of the right T8–9 facet joint taken after capsular denudement, including removal of the facet joint capsule. **(A)** Image of inferior lateral margin of the right T8–9 facet joint showing the lower margin of the inferior articular process (IAP) at 12 o'clock and the transverse process (TP) at 6 o'clock. **(B)** Incrementally more cephalic view (relative to Fig. 4A) of the inferior lateral margin of the right T8–9 facet joint. **(C)** Image of inferior lateral margin of the right T8–9 facet joint, moving incrementally more cephalic along the lateral facet joint margin compared to Fig. 4B. The IAP of the facet joint is shown at 12 o'clock, while the superior margin of TP is now shown at 9 o'clock. **(D)** Incrementally more cephalic view (relative to Fig. 4C) of the right T8–9 facet joint visualizing the superior lateral margin of the facet joint, where the TP of the level is no longer visible. The superiormost aspect of the IAP is shown at 12 o'clock. Note that at approximately 4 o'clock is a circular region where soft tissue superficial to the neural foramen has been denuded, such that slightly deeper tissues (still superficial to the neural foramen) can be visualized. In this case, no additional soft tissue was removed from this region to avoid injury to the neural foramen and potential nerve root damage.

## 3 Case reports

### 3.1 Patient outcomes

Two patients who previously underwent ETFJD at a single tertiary academic medical center agreed to participate as a part of a larger IRB-approved research study (IRB #00138414) designed to assess outcomes of endoscopic joint denervation procedures. These patients had clinically-suspected thoracic facet joint pain based on history, physical examination, and imaging findings, supported by the results of thoracic medial branch blocks. Patient-reported outcomes were obtained via a standardized telephone follow-up assessment, which captured (1) 7-day average pain scores using an 11-point numerical rating scale (NRS); (2) Patient Global Impression of Change (PGIC) scored on a 7-point scale; (3) satisfaction on a 5-point Likert scale (where scores of 1 and 5 represent “very dissatisfied” and “very satisfied”, respectively); (4) Pain and Sleep Questionnaire three-item index (PSQ-3); (5) current and baseline daily opioid use; (6) baseline use of anxiety and/or depression medications; (7) duration of thoracic spine pain prior to ETFJD; and (8) current smoking status. Baseline demographics and clinical characteristics were collected retrospectively from electronic medical records and included (1) age; (2) sex; (3) BMI; (4) ETFJD procedure date, laterality, and treatment levels; (5) number of prior thoracic medial branch RFA procedures; (6) prior fusion surgery; and (7) history of scoliosis and/or spondylolisthesis.

Patients treated with bilateral ETFJD procedures reported outcomes for pain intensity, PGIC, and satisfaction separately for each side at follow-up. No procedural complications or serious adverse events were reported during outcome collection or documented in the electronic medical record. Individual case descriptions are presented below, and patient characteristics and outcome measures are summarized in Table 1.

**Table 1:** Summary of case presentations.

Variable	Patient 1	Patient 2
**Age**	29	43
**Sex**	Male	Female
**BMI (kg/m^2^)**	21.5	22.9
**Current smoking**	No	No
**Pain duration in years**	5	5
**Prior fusion**	No	No
**History of scoliosis**	Yes	No
**History of spondylolisthesis**	No	Yes
**Baseline anxiety and/or depression medication**	Yes	Yes
**Procedure laterality**	Bilateral	Unilateral
**Medial branch level(s) treated**	T10, T11, T12	T3, T4, T5
**Baseline NRS**	9	9
**Follow-up NRS (left/right, if applicable)**	2/7	0
**PGIC**	7/7	7
**Satisfaction**	5/5	5
**PSQ-3**	0	0
**Opioid use at baseline**	Yes	Yes
**Opioid use at follow-up**	No	Yes

NRS = numerical rating scale; PGIC = Patient Global Impression of Change; PSQ-3 = Pain and Sleep Questionnaire three-item index.

### 3.2 Case 1

A 29-year-old male with Scheuermann's kyphosis and thoracolumbar scoliosis presented with chronic bilateral lower thoracic pain that had failed to respond to approximately 5 years of conservative care. History, physical examination, and imaging were consistent with possible facetogenic pain. Diagnostic medial branch blocks were performed, and following positive results on two separate occasions, the patient underwent bilateral thoracic medial branch RFA at T10, T11, and T12 by another physician. Unfortunately, this resulted in only partial and short-lived benefit. Given the history of dual positive medial branch blocks but transient relief with RFA, we offered ETFJD at the same levels when he presented to our clinic. He elected to proceed with ETFJD and underwent this procedure. His baseline NRS pain score was 9. At 14 months follow-up, he reported asymmetric improvement with left-sided pain reduced to 2 (78%) and right-sided pain reduced to 7 (22%). Citing the large magnitude of pain reduction on his left side, he reported maximal satisfaction and global improvement on the PGIC with scores of 5 and 7, respectively. A PSQ-3 score of 0 suggested pain had a minimal role in sleep interference at follow-up. He had not been consuming opioids at baseline but had initiated use at follow-up due to the ongoing right-sided pain.

### 3.3 Case 2

A 43-year-old female presented with chronic right-sided upper thoracic pain that had not improved following approximately five years of conservative care. History, physical examination, and imaging were consistent with possible facetogenic pain. Following dual positive diagnostic medial branch blocks, the patient underwent two thoracic medial branch RFA procedures at T3, T4, and T5 by another physician, each with only transient benefit. Given the history of dual positive medial branch blocks but transient relief with RFA, we offered ETFJD at the same levels when she presented to our clinic, and she elected to proceed with ETFJD. Her baseline NRS pain score was 9. When outcomes were collected at 18 months post-ETFJD, her pain had resolved completely (100%), and she reported marked global improvement with a PGIC score of 7, a satisfaction score of 5, and PSQ-3 score of 0. She had been consuming opioid pain medication at baseline, which had been discontinued at follow-up.

## 4 Discussion

We report a novel technique for thoracic facet joint denervation, using a uniportal endoscopic approach that allows for direct visualization of articular branches transiting to the lateral aspect of the facet joint, regardless of their origin from either the primary dorsal ramus or the proximal medial branch. In addition, the lateral aspect of the facet joint was denuded, such that capsular tissue no longer remained. As such, the present technique combines the advantages of joint denervation by articular branch transection with the advantages of capsular tissue removal. The principles and value of articular branch interruption for joint pain reduction or elimination have been described extensively [13], [14], [15], [16]. Removal of capsular tissue has been theorized to reduce the chances of functional re-innervation of the facet joint as the peripheral nerve endplate receptors within the joint capsule are no longer present, making regeneration unlikely [12]. Additionally, denuding the lateral facet joint of its capsular tissue unavoidably disrupts the articular branch, such that direct visualization of the nerve is not strictly necessary. This technique allows careful avoidance of the exiting spinal nerve via direct visualization, such that nerve transection and capsular debridement can be performed with reduced risk of motor nerve injury compared to RFA techniques that rely only on fluoroscopic electrode placement based on bony landmarks. Notably, this technique avoids entry into the neural foramen, so complete joint denervation may not be possible in all cases, as anatomical variations have been described at middle and lower thoracic levels wherein the articular branch transits to the anterior aspect of the joint capsule directly from the dorsal ramus of the spinal nerve proximal to its exit from the neuroforamen [2], [5].

Additionally, we provide two case reports in which this technique for ETFJD was applied. The two patients included in the present description reported clinically meaningful NRS pain score reductions of ≥50% and reported PGIC scores consistent with “very much improved” at ≥1 year follow-up. No procedural complications occurred. These findings do not provide statistical evidence of treatment effectiveness, but do justify future outcome research.

To date, published literature on endoscopic medial branch transection or coagulation has primarily focused on lumbar applications, including techniques variably described as endoscopic “rhizotomy,” “neurotomy,” “neurectomy,” or “denervation.” The current literature demonstrates durable improvements in pain and disability in selected cohorts [10], [11], [17]. In contrast, thoracic facet interventions have been more extensively evaluated using percutaneous fluoroscopically guided radiofrequency ablation techniques, with heterogeneous outcomes across procedural approaches and patient populations [18], [19], [20], [21], [22], [23]. The complex and level-dependent anatomical variability of articular branch origin from the thoracic spinal dorsal ramus and/or medial branch nerve may contribute to incomplete neural capture during fluoroscopically guided percutaneous lesioning, particularly at mid-thoracic levels where anatomical variability appears to be greatest [2], [4], [5], [7]. For these reasons, we believe that further investigation of the safety and effectiveness of our proposed ETFJD technique is warranted.

## 5 Conclusion

We describe a novel technique for endoscopic thoracic facet joint denervation in the context of updated neuroanatomical evidence related to facet joint innervation and also describe two case reports. Formal investigation of clinical outcomes associated with this treatment strategy for well-selected patients with refractory thoracic facet joint pain is warranted.

## Consent

Patient consent was obtained for disclosure of the case details.

## Funding

This work was supported by the Skaggs Clinical Spine Research Seed Grant Program at the 10.13039/100007747University of Utah and a research grant from the June Morris and Frendt Family Foundation.

## Conflict of interest statement

Zachary L. McCormick, MD serves on the Board of Directors of the International Pain and Spine Intervention Society (IPSIS), has research grants from Avanos Medical, Boston Scientific, DiscGenics, Presidio Medical, Saol Therapeutics, Spine Biopharma, SPR Therapeutics, and Stratus Medical (paid directly to the University of Utah), and previous consultancies with Avanos Medical, Saol Therapeutics, Stryker, and OrthoSon (all ended). Aaron Conger, DO has received research grants and is a consultant for Stratus Medical. Mark Mahan, MD has research grants from Boston Scientific, Axogen, and Renerva (all paid to the University of Utah) and consulting contracts with Arthrex, joimax, and Spineology.
